# Identification of novel pig and human immunoglobulin G-binding proteins and characterization of the binding regions of enolase from *Streptococcus suis* serotype 2

**DOI:** 10.1186/s13568-020-01042-2

**Published:** 2020-06-01

**Authors:** Quan Li, Yang Fu, Genglin Guo, Zhuohao Wang, Wei Zhang

**Affiliations:** 1 MOE Joint International Research Laboratory of Animal Health and Food Safety, Key Lab of Animal Bacteriology of Ministry of Agriculture, OIE Reference Lab for Swine Streptococcosis, College of Veterinary Medicine, Nanjing Agricultural University, Nanjing, China; 2 College of Veterinary Medicine, Yangzhou University, Yangzhou, 225009 China; 3 Jiangsu Co-innovation Center for the Prevention and Control of Important Animal Infectious Diseases and Zoonoses, Yangzhou, 225009 China

**Keywords:** *Streptococcus suis*, IgG-binding proteins, Surface proteins, Interactions, Enolase

## Abstract

*Streptococcus suis*, a major emerging pathogen in swine and humans, expresses immunoglobulin G (IgG)-binding proteins (IBPs), which contribute to the ability of organism to evasion of host immune system. The objective of this study was to identify novel pig IgG (pIgG) and human IgG (hIgG)-binding proteins and characterize the binding regions of enolase from *Streptococcus suis* serotype 2 (*S. suis* 2). Here, four pIgG-binding proteins (pIBPs) and five hIgG-binding proteins (hIBPs) were identified from *S. suis* 2 surface proteins by 2D-Far-western blot assays. All the newly captured proteins were expressed and further confirmed their binding activity to pIgG or hIgG by Far-western blot and dot blot. In addition to previously identified factor H, fibronectin, collagen, fibrinogen, plasminogen and laminin, we also found that both pIgG and hIgG can specifically interact with enolase. Binding assays indicated that interactions of *S. suis* 2 enolase with pIgG and hIgG is primarily mediated by the enolase C-terminal portion (Enolase-C, a.a. 142–432). We found that hIgG exhibited stronger binding ability to Enolase-C than pIgG. Further analysis of the C-terminal regions of enolase (Enolase-C1 and Enolase-C2) suggested that the C-terminus possessed two different binding domains with distinct host IgG proteins. Strikingly, we confirmed that pIgG interacted with the Enolase-C1 (a.a. 142–271) and hIgG interacted with the Enolase-C2 (a.a. 271–432). These observations of enolase provide interesting insights in the pathogenesis of *S. suis* infection.

## Introduction

*Streptococcus suis* has emerged as an important zoonotic agent attributed in causing diseases such as arthritis, endocarditis, meningitis, and septicemia in pigs (Feng et al. 2010; Staats et al. 1997). It is also responsible for a series of serious infections associated with meningitis, septicemia, and acute death in humans (Gottschalk et al. 2007). Among the 35 serotypes (types 1 to 34, and 1/2) depended on the difference of capsular antigens, *Streptococcus suis* serotype 2 (*S. suis* 2) is the most virulent and prevalent one, responsible for most humans and swine infection cases in Asia and North America. Globally, serotypes 9 and 7 are also the predominant *S. suis* serotypes involved in pig infections (Goyette-Desjardins et al. 2014). In the past, several approaches have been used to explore protective antigens for preventing *S. suis* infection. However, no ideal therapeutics or vaccine against *S. suis* infections is available thus far, although some researches showed homologous protection (Baums et al. 2009; Feng et al. 2014; Fittipaldi et al. 2012). To date, a multitude of virulence factors have been reported, such as MRP Li et al. (2017b; Wisselink et al. 2001), SLY (Du et al. 2013; Jacobs et al. 1996), EF (Wisselink et al. 2001), enolase (Esgleas et al. 2009; Feng et al. 2009; Zhang et al. 2009), HtpsC (Li et al. 2015a), and HP0272 (Chen et al. 2010; Pian et al. 2012), but the pathogenesis of *S. suis* infections remains poorly understood (Fittipaldi et al. 2012).

Immunoglobulin G (IgG) is the major antibody of humoral immunity found in extracellular fluid and blood allowing it to protect the host tissues from infection. IgG-mediated binding of pathogen allows their recognition by phagocytic immune cells that results in pathogen elimination. However, a variety of pathogens express surface IgG-binding proteins (IBPs) to recruit IgG to evade the host defences in a non-immune mechanism (Bessen and Fischetti 1990; Blumenfeld et al. 1990). IBPs play important roles in the capacity of bacterial pathogens to evade IgG-mediated phagocytosis by interfering with complement consumption, phagocytosis or opsonization (Serhir et al. 1993; Widders et al. 1989). The presence of IBPs have been reported on some pathogenic streptococci (Nobbs et al. 2009). A number of IBPs in streptococci of groups A, B and C shown to be important virulence factors, and contribute to escape detection by the immune system, including SfbI, Sib35, SibA, protein H, Sir, M protein, M-like proteins, and Mrp of *S. pyogenes*; Lzp of *S. agalactiae*; Protein G, FOG, MAG, and DemA of *S. dysgalactiae*. Although a multitude of IBPs were found in various bacteria, the research of *S. suis* IBPs was neglected for years. Thus far, only a 52-kDa IgG-binding protein enolase was identified and characterized (Serhir et al. 1993, 1995). Therefore, large scale identification of the IBPs of *S. suis* 2 that interact with IgG will provide valuable insights into the mechanism of *S. suis* induced pathogenesis. Far-western blot assay is an efficient method to characterize protein–protein interactions that can be used to identify specific interacting proteins in complex mixture samples. In our previous study, it has been successfully used for the identification of factor H-, laminin- and fibronectin-binding proteins of *S. suis* 2 (Li et al. 2015b, 2017c). In this work, four pIBPs and five hIBPs, including enolase, Peptidoglycan-binding protein LysM (LysM), Pyruvate kinase (Pyk), Lactate dehydrogenase (LDH), Fructose-bisphasphate aldolase (FBA), and 3-Ketoacyl-ACP reductase (KAR) were identified from *S. suis* 2 surface proteins using this approach.

Surface proteins on Gram-positive bacteria are often multifunctional molecules with two or more independent functions. Enolase, a conserved surface protein, is multifunctional in its enzymatic activity and can bind different host components found in body secretions, which include plasminogen (Esgleas et al. 2008), fibronectin (Esgleas et al. 2008), fibrinogen (Pian et al. 2015), laminin (Li et al. 2015b; Zhang et al. 2014b), factor H (Li et al. 2017c), and collagen (Zhang et al. 2014a). Previous studies indicated that *S. suis* enolase can facilitate the adherence to and invasion of host cells (Esgleas et al. 2008; Zhang et al. 2009). Sun et al. had reported that enolase can significantly disrupt the blood–brain barrier integrity by inducing IL-8 release (Sun et al. 2016). Additionally, Feng et al. demonstrated that enolase can function as a protective antigen against *S. suis* 2 infection (Feng et al. 2009). In the present study, we also identified that both pIgG and hIgG can specifically interact with enolase. The interactions of enolase specific regions with pIgG and hIgG was further evaluated. We found that the binding region of enolase to pIgG and hIgG is primarily mediated by Enolase-C. Furthermore, binding assays confirmed that pIgG interacted with the Enolase-C1 and hIgG interacted with the Enolase-C2. Taken together, these observations of enolase improved our understanding of the pathogenesis of *S. suis* 2 infection.

## Materials and methods

### Bacterial strains and culture conditions

The *S. suis* 2 strain ZY05719 is one of the representative Chinese virulent strains and isolated from a diseased pig during an outbreak in Sichuan, China. The bacteria were maintained in Todd Hewitt Broth (THB; Becton Dicksinson, USA) liquid or agar media at 37 °C and harvested at the mid-exponential phase. *E. coli* strains BL21 (DE3) were maintained in Luria–Bertani Broth (LB; OXOID) liquid medium or plated on LB agar at 37 °C. When necessary, 100 µg/ml ampicillin (Amp; Sigma) was used for screen the *E. coli* transformants. The pET-32a vectors were used for protein expression.

### Preparation of cell wall and extracellular proteins

Cell wall-associated proteins were prepared according to our previous study (Li et al. 2015b, 2017c). Briefly, the growth points of the bacteria were evaluated by measuring the OD600 at 1-h intervals using a spectrophotometer. The bacteria samples from middle stage of exponential phase (OD600 = 0.8) were separated by centrifugation at 4 °C, and then resuspended in solution buffer containing 30 mM Tris–HCl (pH 7.5), 3 mM MgCl_2_, 125 U/ml mutanolysin, 25% sucrose, and incubated for 90 min at 37 °C. The cell lysate was centrifuged at 4 °C for 10 min and the supernatant were precipitated in 10% trichloroacetic acid (TCA) for 30 min at 4 °C. The proteins were washed two times with 10 ml chilled acetone to remove the residual TCA, and then dried the pellet in air.

Extracellular proteins were prepared as we recently described (Li et al. 2015b). In short, culture supernatant was separated by centrifugation at 4 °C and then filtered twice through a 0.22 μm membrane filters. Then, the supernatant were precipitated in 10% TCA for 30 min at 4 °C. The proteins were washed two times with 10 ml chilled acetone to remove the residual TCA, and then dried the pellet in air. The concentration of cell wall and extracellular proteins was determined by a BCA protein assay kit (Beyotime).

### Identification of pIBPs and hIBPs by 2D-Far-western blot

The 2D-Far-western blot experiment was carried out according to our previous study (Li et al. 2015b, 2017c). In brief, the cell wall and extracellular protein samples (~ 200 µg) were solubilized in a 250 µl solution buffer containing 0.2% w/v DTT, 2 M thiourea, 7 M urea and 2% w/v CHAPS at 25 °C for 30 min. The insoluble components were removed with the 2-D Clean-up Kit (GE Healthcare). Subsequently, samples were resuspended in 250 µl rehydration solution containing 0.2% w/v DTT, 2% w/v CHAPS, 0.002% w/v bromophenol blue, 7 M urea, 2 M thiourea, and 0.5% v/v IPG buffer. And then loaded into immobilized pH gradient strips (13 cm; pH 4–7; GE Healthcare) for isoelectric focusing (IEF) analysis.

For Far-western blot analysis, the cell wall and extracellular proteins were subjected to 12% SDS-PAGE, and then transferred onto polyvinylidene difluoride (PVDF) membranes (Merck Millipore). The membranes were blocked for 12 h with 5% w/v skimmed milk diluted with Tris-buffered saline with Tween 20 (TBST) at 4 °C. After discarding the blocking buffer, membranes were incubated with pIgG (Sigma; 20 µg/ml) or hIgG (Sigma; 20 µg/ml) for 24 h at 4 °C. At the same time, a membrane incubated with 20 µg/ml BSA was used as a negative control, followed by three washes with the washing buffer TBST and incubated with HRP conjugated staphylococcal protein A (SPA) (Boster; 1:3000 dilution) for 1 h at 37 °C. After three washes, the positive proteins were developed using 3,3′-diaminobenzidine (DAB; Tiangen, China).

### Identification of positive spots by MALDI-TOF-MS

Identification of the positive proteins were carried out according to our previous study (Li et al. 2015b). In brief, the detected protein spots were excised from the gels for digestion and subjected to MALDI-TOF–MS analysis. Peptide mass fingerprinting data were analyzed using the MASCOT server (http://www.matrixscience.com). Peptides with a rank of 1 in the MASCOT search were considered significant and used for the combined peptide score.

### Expression and purification of recombinant IBPs and enolase truncations

To express recombinant pIBPs and hIBPs in *E. coli*, ZY05719 genomic DNA was used for PCR with the primers described in Table 1. *S. suis* IBPs (Enolase, LysM, Pyk, LDH, FBA, KAR) and enolase truncations (Enolase-N, Enolase-C, Enolase-C1, Enolase-C2) were amplified, and the PCR products were inserted into the pMD19-T and then cloned into the pET-32a vector using BamHI/XhoI or BamHI/EcoRI restriction enzymes. The pET-32a plasmids with inserts were screened by PCR amplification using primers. The cloned gene sequences were confirmed by direct DNA sequencing. Afterwards, the positive clone was transformed into *E. coli* strain BL21 (DE3) for expression. Bacteria were induced with 1 mM IPTG for 4 h at 37 °C when the OD600 was between 0.5 and 0.6. Bacterial cells were harvested by centrifugation at 4 °C. The recombinant His-tagged proteins were purified by Ni-chelating affinity gel (GE Healthcare) according to the instruction manual. After passage through a 0.22-µm filter (Millipore), purified recombinant proteins were stored at − 80 °C. The concentration of recombinant proteins were confirmed by BCA protein assay kit (Beyotime).

**Table 1:** Primers used in this study

Primers	Sequences (5′-3′)^a^	Length of PCR products (bp)	Function
Enolase-F	CGCGGATCCATGTCAATTATTACTGATGTT	1308	The ORF of enolase
Enolase-R	CCGCTCGAGTTATTTTTTCAAGTTGTAGAA		
LysM-F	CGCGGATCCCCACAACATATGCGTCGCAAG	990	The ORF of LysM
LysM-R	CCGGAATTCCACATGATCGTAGTGGTTTTC		
Pyk-F	CGCGGATCCGCAACCCTTGGTCCAGCGGTA	1476	The ORF of Pyk
Pyk-R	CCGCTCGAGTTATACTGTACGAACACGCATAGT		
LDH-F	CGCGGATCCACTGCAACTAAACAACACAAA	978	The ORF of LDH
LDH-R	CCGCTCGAGTTAGTTTTTTACACCAGCTGCAAT		
FBA-F	CGCGGATCCACAAACAACCTTGAGTGGACT	798	The ORF of FBA
FBA-R	CCGCTCGAGCGCTGAACCGAATACGTCGAT		
KAR-F	CGCGGATCCCTGCATGGTCAAGCCTCCGTC	732	The ORF of KAR
KAR-R	CCGCTCGAGGTAACAGGTTCAAGTCGAGGA		
Enolase-N-F	CGCGGATCCATTACTGATGTTTACGCTCGC	435	The ORF of enolase-N
Enolase-N-R	CCGCTCGAGTTAGTTCATCATTGGAGTTGGCAA		
Enolase-C-F	CGCGGATCCTTGCCAACTCCAATGATGAAC	873	The ORF of enolase-C
Enolase-C-R	CCGCTCGAGTTAGTTGTAGAATGAGTTCAAGCC		
Enolase-C1-F	CGCGGATCCTTGCCAACTCCAATGATGAAC	390	The ORF of enolase-C1
Enolase-C1-R	CCGCTCGAGTGTACGAACAGCAGCGCCTTC		
Enolase-C2-F	CGCGGATCCACATCTGCAGAACAAATCGAC	486	The ORF of Enolase-C2
Enolase-C2-R	CCGCTCGAGTTAGTTGTAGAATGAGTTCAAGCC		

^a^The underlined sequences are restriction enzyme sites

### Binding assays by Far-western blot

The Far-western blot analysis of recombinant IBPs and enolase truncations to pIgG or hIgG was carried out according to our previous study (Li et al. 2015b, 2017c). In brief, recombinant proteins (~ 10 µg) and casein were subjected to 12% SDS-PAGE, and then transferred onto PVDF membranes. The membranes were blocked for 12 h with 5% w/v skimmed milk diluted with TBST at 4 °C. After discarding the blocking buffer, membranes were incubated with pIgG (Sigma; 20 µg/ml) or hIgG (Sigma; 20 µg/ml) for 24 h at 4 °C. Subsequently, the membranes were washed three times with the washing buffer TBST and incubated with HRP conjugated SPA (Boster; 0.2 µg/ml) for 1 h at 37 °C. After three washes, the positive proteins were developed using 3,3′-diaminobenzidine (DAB; Tiangen, China). Casein was used as a negative control for non-specific binding to IgG.

### Binding assays by dot blot

The dot blot analysis of recombinant IBPs to pIgG or hIgG was performed as previously described (Li et al. 2017a; Lu et al. 2008). Equal volumes (3 μl) of recombinant proteins were each spotted in duplicate onto the methanol-activated PVDF membranes. The membranes were air dried for 5 min and blocked for 12 h with 5% w/v skimmed milk diluted with TBST at 4 °C. After discarding the blocking buffer, membranes were incubated with pIgG (Sigma; 20 µg/ml) or hIgG (Sigma; 20 µg/ml) for 24 h at 4 °C. Subsequently, the membranes were washed three times with TBST and incubated with HRP conjugated SPA for 1 h at 37 °C. After three washes, dots were developed using 3,3′-diaminobenzidine (DAB; Tiangen, China). Casein was used as a negative control for non-specific binding to IgG. The membrane with HRP conjugated SPA alone were used as a blank control.

## Results

### Identification of novel *S. suis* 2 pIBPs and hIBPs by proteomics and Far-western blot

**Fig. 1:**
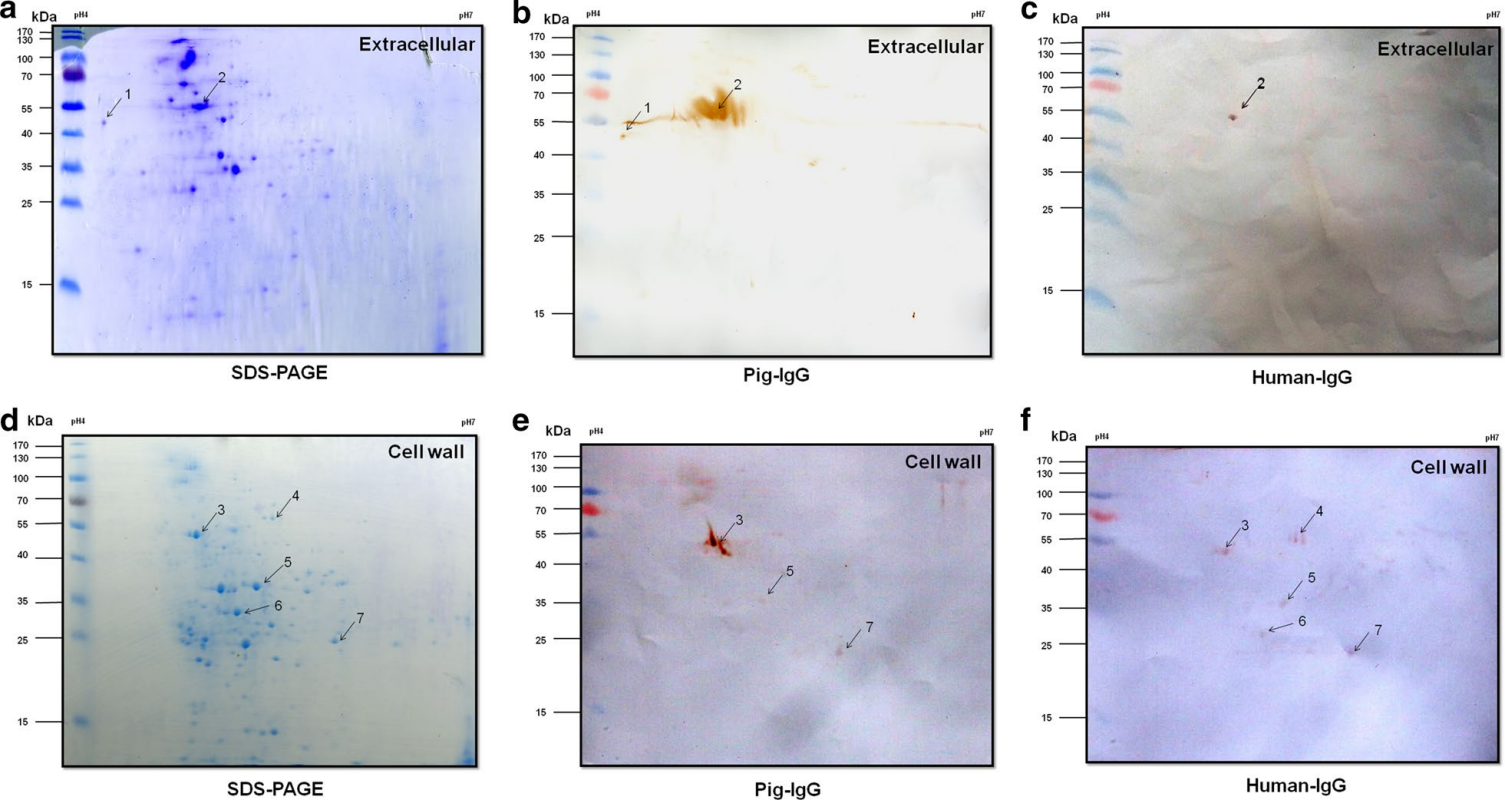
2-DE profiles and Far-western blot to identify the affinity of pIgG and hIgG interactions with *S. suis* 2 cell wall and extracellular proteins. The cell wall and extracellular proteins of *S. suis* 2 were loaded into immobilized pH gradient strips for IEF analysis and by SDS-PAGE in the second dimension. The 2-DE gels were transferred onto PVDF membranes and incubated with pIgG or hIgG. Arrows indicate pIBPs or hIBPs detected with HRP conjugated SPA. **a** 2-DE gel of *S. suis* 2 extracellular proteins. **b** Far-western blot of extracellular proteins incubated with pIgG. **c** Far-western blot of extracellular proteins incubated with hIgG. **d** 2-DE gel of *S. suis* 2 cell wall proteins. **e** Far-western blot of cell wall proteins incubated with pIgG. **f** Far-western blot of cell wall proteins incubated with hIgG

The cell wall and extracellular proteins of *S. suis* 2 were subjected to 2D SDS-PAGE and transferred onto PVDF membranes for Far-western blot analysis. According to our previous study (Li et al. 2015b), at least 200 Coomassie blue stained protein spots were identified on the two-dimensional electrophoresis (2-DE) gels (Fig. 1a and d, left panels). Only the IBPs, which were clearly observed on the 2-DE gels, consistent with the positive response spots on the membranes, were selected. In this way, two pIBPs (Fig. 1b) and one hIBP (Fig. 1c) from *S. suis* 2 extracellular proteins were detected. By using the same method, three pIBPs (Fig. 1e) and five hIBPs (Fig. 1f) in cell wall proteins were identified. No positive response spots were detected when the membrane was incubated with BSA (a negative control). The detected protein spots were manually excised from the gels and subjected to analysis by MALDI-TOF–MS. Finally, a total of six IBPs, including four pig pIBPs and five hIBPs, were identified from *S. suis* 2 surface proteins by 2D-Far-western blot immunoassay. All the identified IBPs except enolase were discovered for the first time. Three proteins enolase, FBA, and KAR displayed binding abilities to both pIgG and hIgG. The data of the positive spots are listed in Table 2.

**Table 2:** Identification of the potential human and pig IgG-binding proteins by MALDI-TOF–MS

Spot no.^a^	Identified protein	Accession no.	Theoretical pI/MW^b^	Experimental pI/MW	MASCOT score	Coverage (%)
1	Peptidoglycan-binding protein LysM	AKG39650.1	4.52/37564	4.2/45000	117	63
2, 3	Enolase	AKG40756.1	4.66/47095	4.7/50000	143	36
4	Pyruvate kinase	AKG39916.1	5.12/54630	5.2/57000	351	54
5	Lactate dehydrogenase	AKG40380.1	5.05/35422	5.0/37000	258	42
6	Fructose-bisphasphate aldolase	AKG39743.1	4.90/31155	4.9/32000	375	44
7	3-Ketoacyl-ACP reductase	AKG41028.1	5.53/25589	5.7/25000	263	29

^a^Spot numbers correspond to those indicated in Fig. 1

^b^Theoretical pI and MW were calculated using the Compute pI/Mw server (http://web.expasy.org/compute_pi/)

### Confirmation of the binding activity of pIBPs and hIBPs

**Fig. 2:**
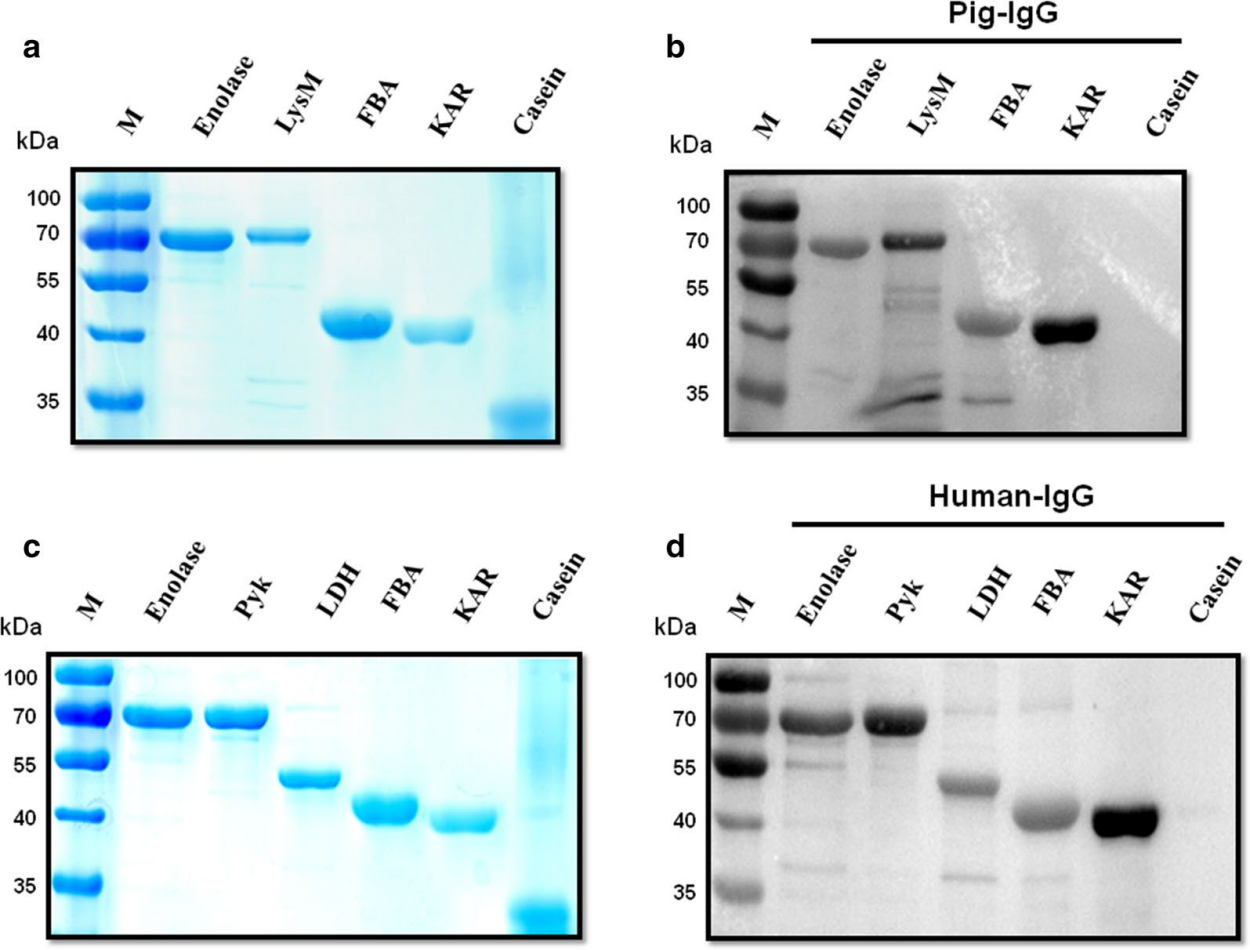
Identification the binding of pIgG or hIgG to IBPs of *S. suis* 2 by Far-western blot. SDS-PAGE (**a**, **c**) and Far-western blot analysis (**b**, **d**) of the *S. suis* 2 recombinant IBPs. Recombinant proteins (Enolase, LysM, Pyk, LDH, FBA, KAR) and casein were subjected to SDS-PAGE, then transferred onto PVDF membranes and incubated with pIgG or hIgG. Bound pIgG or hIgG was recognized with HRP conjugated SPA. Casein was used as a negative control for non-specific binding to IgG

To prove that the identified proteins could interact specifically with pIgG or hIgG, the ligand binding assays was further performed by Far-western blot and dot blot analysis. As shown in Fig. 2a and c, recombinant enolase, LysM, Pyk, LDH, FBA, and KAR proteins were purified successfully by Ni-chelating affinity gel. The results indicated that recombinant enolase, LysM, FBA, and KAR were able to interact with pIgG (Fig. 2b). The hIgG-binding activity of recombinant enolase, Pyk, LDH, FBA, and KAR were also detected by Far-western blot analysis (Fig. 2d). As expected, pIgG and hIgG failed to bind to the negative control protein casein. These data suggested that all the identified IBPs of *S. suis* 2 bind specifically to IgG.

**Fig. 3:**
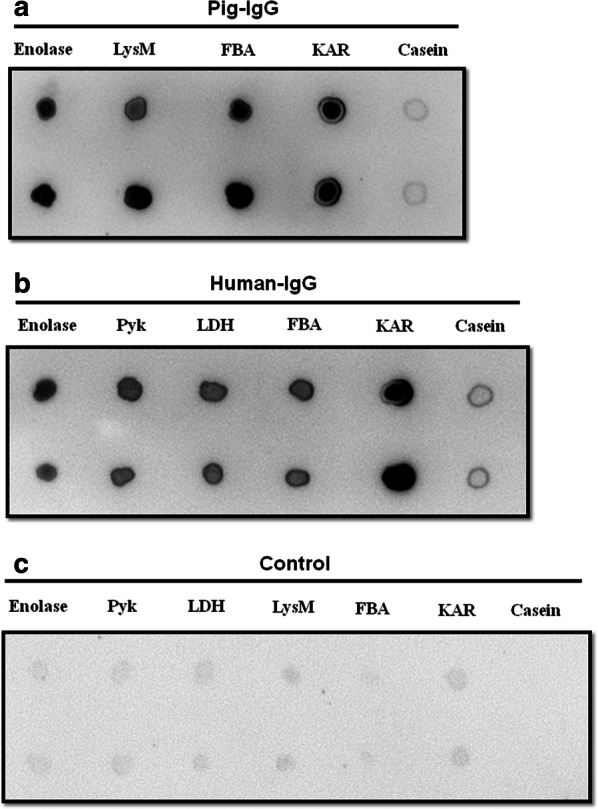
Identification the binding of pIgG or hIgG to IBPs of *S. suis* 2 by dot blot. Recombinant pIBPs (**a**) and hIBPs (**b**) were spotted onto the methanol-activated PVDF membranes and incubated with pIgG or hIgG. Bound pIgG or hIgG was recognized with HRP conjugated SPA. Casein was used as a negative control for non-specific binding to IgG and the membrane with HRP conjugated SPA alone were used as a blank control

Furthermore, the interaction of recombinant IBPs with pIgG or hIgG was also evaluated by dot blot analysis. Our data confirmed that recombinant captured IBPs showed strong binding to pIgG (Fig. 3a) and hIgG (Fig. 3b), while the casein, a negative control protein, almost had no specific bind to pIgG or hIgG. Under similar assay conditions, no positive response was observed when the membrane was only incubated with the secondary antibody (Fig. 3c). Dot blot analysis demonstrated all the captured *S. suis* 2 IBPs could interact with IgG, which is in general consistency with the result of the Far-western blot assay.

### Identification of enolase regions that interact with pIgG and hIgG

**Fig. 4:**
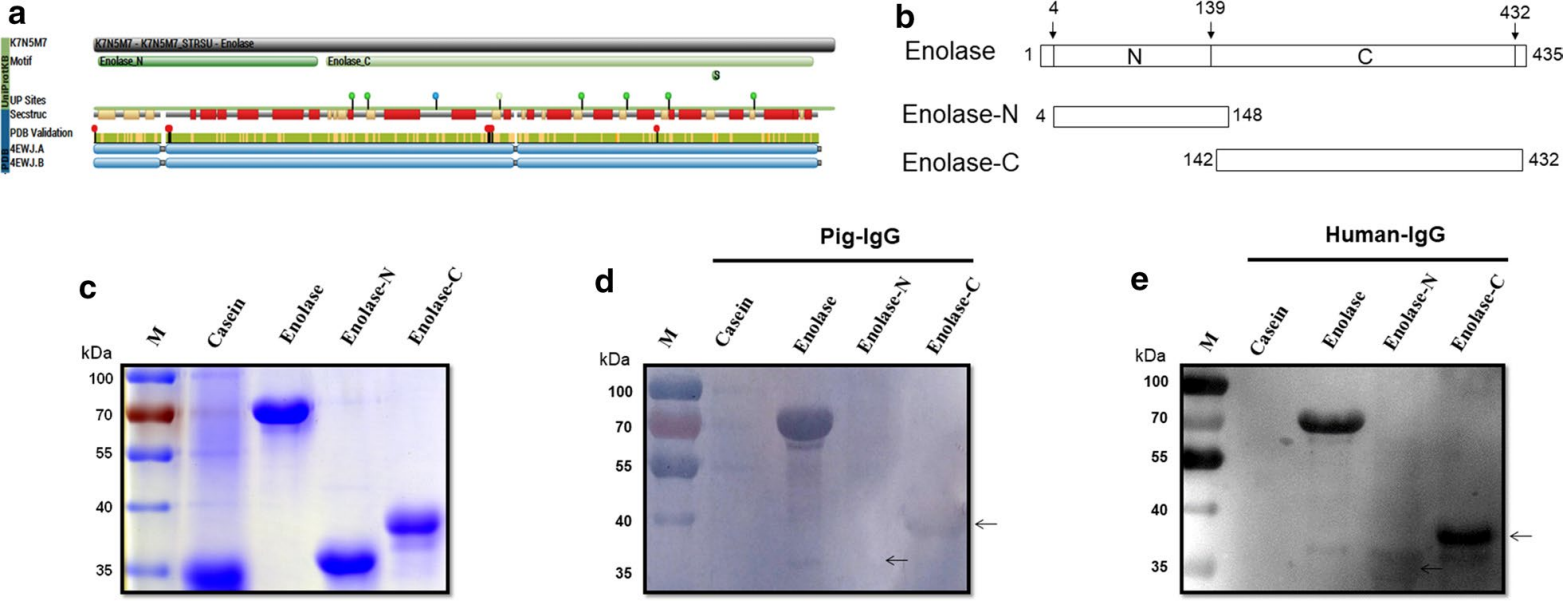
Interaction of *S. suis* 2 enolase and its regions with pIgG or hIgG. **a** The enolase putative domain predicted by the PDB database. **b** Schematic representation of two recombinant truncations of enolase (Enolase-N and Enolase-C). The truncations of enolase were engineered mainly according to the PDB database. **c** SDS-PAGE analysis of recombinant enolase, Enolase-N, and Enolase-C. **d** Far-western blot analysis of enolase and its truncations incubated with pIgG. **e** Far-western blot analysis of enolase and its truncations incubated with hIgG

Enolase is a multifunctional surface protein of *S. suis*, which could bind both pIgG and hIgG, as well as a variety of host component proteins. However, the binding subdomain of enolase to pIgG or hIgG remains unclear. According to the PDB database, the enolase putative domain can be divided into N-terminal and C-terminal portion (Fig. 4a). In an attempt to determine the pIgG- and hIgG-binding regions of enolase, we engineered two recombinant truncations of enolase mainly according to the PDB database, designated enolase N-terminal portion (Enolase-N, a.a. 4–148) and enolase C-terminal portion (Enolase-C, a.a. 142–432), with an overlapping portion of 7 amino acids (Fig. 4b). As shown in Fig. 4c, recombinant Enolase-N and Enolase-C were purified successfully by Ni-chelating affinity gel. The pIgG-binding activity of recombinant Enolase-C was detected by Far-western blot analysis, while Enolase-N almost had no specific bind to pIgG (Fig. 4d). Furthermore, we detected both Enolase-N and Enolase-C can interact with hIgG, whereas Enolase-C exhibited higher binding activity to hIgG compared with Enolase-N (Fig. 4e). Under similar assay conditions, pIgG and hIgG failed to bind to the negative control protein casein. Our results indicated that interactions of enolase with pIgG and hIgG is primarily mediated by Enolase-C.

### Two distinct regions of the carboxyl terminus bind to different host IgG proteins

**Fig. 5:**
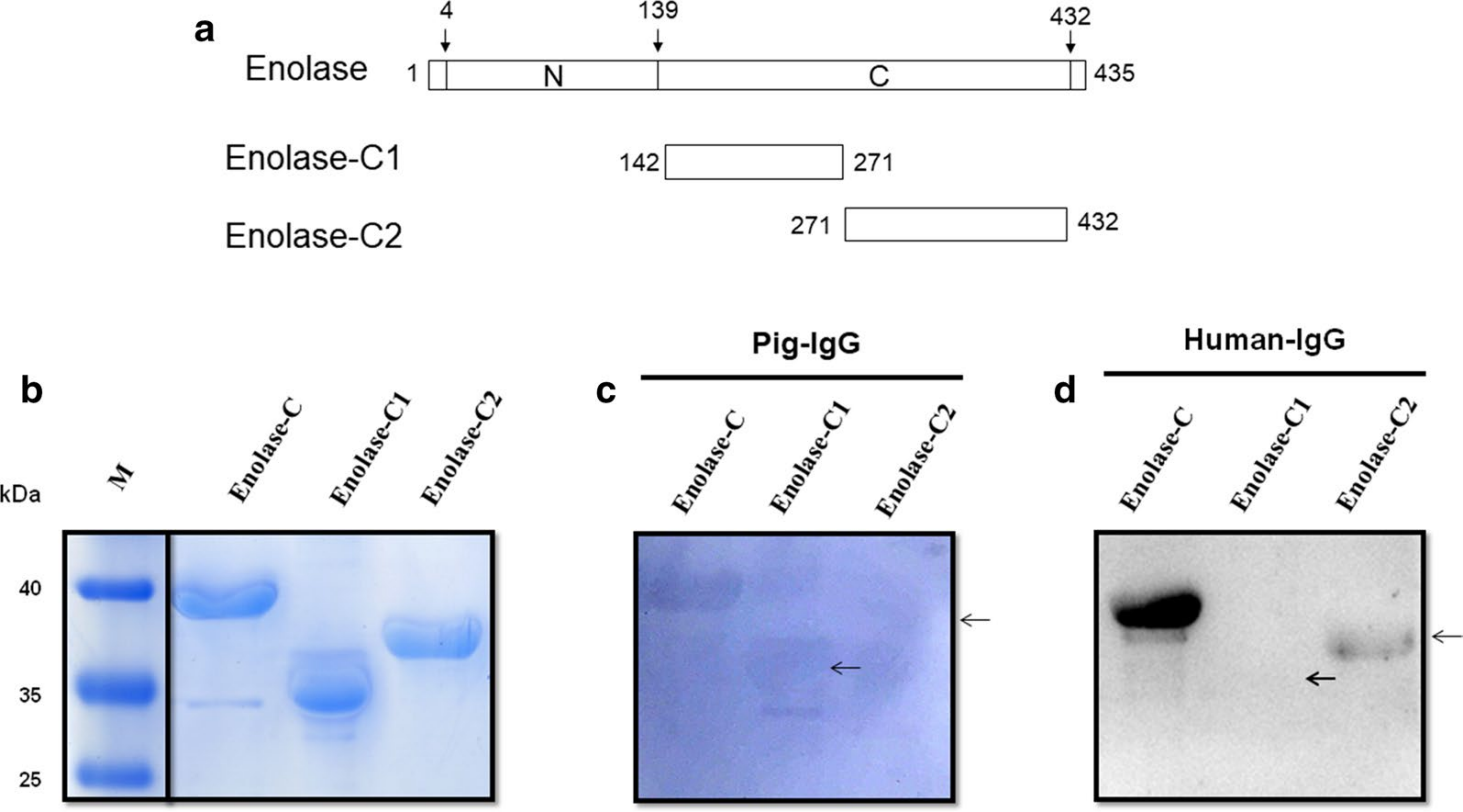
Characterization of enolase truncations that interact with pIgG or hIgG. **a** Schematic representation of two deletion constructs Enolase-C1 and Enolase-C2. **b** SDS-PAGE analysis of recombinant Enolase-C, Enolase-C1, and Enolase-C2. **c** Far-western blot analysis of Enolase-C and its truncations incubated with pIgG. **d** Far-western blot analysis of Enolase-C and its truncations incubated with hIgG

To further determine the pIgG- and hIgG- binding regions of Enolase-C, two recombinant truncations of Enolase-C were constructed, designated Enolase-C1 (a.a. 142–271) and Enolase-C2 (a.a. 271–432) (Fig. 5a). After purification by Ni-chelating affinity gel, SDS-PAGE analysis confirmed that recombinant Enolase-C1 and Enolase-C2 were purified successfully (Fig. 5b). As shown in Fig. 5c and d, the binding activity of hIgG to Enolase-C was stronger than pIgG. The data showed that Enolase-C possessed two different binding domains (Enolase-C1 and Enolase-C2) with distinct host IgG proteins. We found that pIgG were able to interact with Enolase-C1, while we detected no pIgG-binding activity for Enolase-C2 (Fig. 5c). Notably, the hIgG-binding ability of Enolase-C2 was detected, while Enolase-C1 almost had no specific bind to hIgG (Fig. 5d). Of particular note, Enolase-C exhibited stronger binding ability to hIgG than Enolase-C2 (Fig. 5d).

## Discussion

*Streptococcus suis* has emerged as an important zoonotic agent that can be transmitted to humans and is responsible for severe financial losses in the global swine industry. It causes septicemia, arthritis, meningitis, and endocarditis in swine (Lun et al. 2007), and also cause serious injuries such as septicemia, meningitis, permanent hearing loss in humans who come into contact with infected pigs or pork-derived products (Gottschalk et al. 2007). In 1998 and 2005, two major outbreaks of human infection caused by *S. suis* 2 raised enormous public concern in China (Tang et al. 2006; Yu et al. 2006). Strikingly, both outbreaks lead to streptococcal toxic shock syndrome (STSS), which is showed a prevalent feature of acute high fever and high mortality rate despite antibiotic therapy (Sriskandan and Slater 2006).

IgG is the major antibody of humoral immunity found in extracellular fluid and blood. By binding many kinds of pathogens such as fungi, viruses, and bacteria, IgG can protect the host tissues from infection. The ability to interact with IgG in a non-immune reaction is a feature shared by streptococcal groups (Bessen and Fischetti 1990). The main purpose of the present study was to search for novel pIgG and hIgG-binding proteins in *S. suis*. Four pIBPs and five hIBPs were captured from *S. suis* 2 surface proteins by 2D-Far-western blot assays. The captured proteins were further evaluated their binding ability to pIgG or hIgG by Far-western blot and dot blot. In general consistency with the results of 2D-Far-western blot, we found that all the identified proteins were able to interact with pIgG or hIgG. All the identified IBPs except enolase were discovered for the first time. Four proteins enolase, LDH, FBA, and KAR have been reported as extracellular matrix (ECM) binding proteins, of which enolase, LDH, and FBA involved in adherence of *S. suis* 2 (Li et al. 2015b). Additionally, enolase, Pyk, FBA, and KAR have been identified as factor H binding proteins in our recent study (Li et al. 2017c). LysM has been documented as a surface protein contributes to *S. suis* 2 virulence. These data provide important clues of *S. suis* pathogenesis.

Enolase, a glycolytic enzyme of the glycolysis pathway, was identified as a highly conserved immunogenic protein, which is present at the surface of all the described *S. suis* serotypes (Esgleas et al. 2008; Feng et al. 2009). It is also a very highly conserved protein among *streptococcus* species (> 93% homology with other streptococcal enolase) (Esgleas et al. 2008; Jing et al. 2008). Previous studies indicated that enolase plays an important role in the adhesion and pathogenesis of *S. suis* with specifically binding activity to many host components. Zhang et al. (2009) and Feng et al. (2009) had previously demonstrated that enolase could elicit good protection against *S. suis* infection in a mouse model. In this study, we also identified that both pIgG and hIgG can specifically interact with enolase. Hence, characterize the binding regions of enolase that interact with pIgG or hIgG may give insight into the pathogenesis of *S. suis*.

Collectively, we determined that the binding region of enolase to pIgG and hIgG is primarily mediated by Enolase-C (a.a. 142–432). Further results indicated that Enolase-C possessed two different binding domains with distinct host IgG proteins. We found that pIgG were able to interact with the Enolase-C1, while hIgG bind to the Enolase-C2. These data of enolase could contribute to a better understanding of the pathogenesis of *S. suis* induced infection.

## Data Availability

Not applicable.
